# Randomized Comparison of Isosorbide Mononitrate and PGE2 Gel for Cervical Ripening at Term including High Risk Pregnancy

**DOI:** 10.1155/2014/147274

**Published:** 2014-07-01

**Authors:** Kavita Agarwal, Achla Batra, Aruna Batra, Abha Aggarwal

**Affiliations:** ^1^Department of Obstetrics & Gynaecology, Safdarjung Hospital, G-14, 92 Vrindavan Apartment, Gali No. 4, Krishna Nagar, Safdarjung Enclave, New Delhi 110029, India; ^2^NIMS, Delhi 110029, India

## Abstract

*Aims*. Prostaglandin E2 is the most commonly used drug for cervical ripening prior to labour induction. However, there are concerns regarding uterine tachysystole and nonreassuring fetal heart (N-RFH). Isosorbide mononitrate (IMN) has been used successfully for cervical ripening. The present study was conducted to compare the two drugs for cervical ripening at term in hospital. *Methods*. Two hundred women with term pregnancies referred for induction of labour with Bishop score less than 6 were randomly allocated to receive either 40 mg IMN tablet vaginally (*n* = 100) or 0.5 mg PGE2 gel intracervically (*n* = 100). Adverse effects, progress, and outcomes of labour were assessed. *Results*. PGE2 group had significantly higher postripening mean Bishop score, shorter time from start of medication to vaginal delivery (13.37 ± 10.67 hours versus 30.78 ± 17.29 hours), and shorter labour-delivery interval compared to IMN group (4.53 ± 3.97 hours versus 7.34 ± 5.51 hours). However, PGE2 group also had significantly higher incidence of uterine tachysystole (15%) and N-RFH (11%) compared to none in IMN group, as well as higher caesarean section rate (27% versus 17%). *Conclusions*. Cervical ripening with IMN was less effective than PGE2 but resulted in fewer adverse effects and was safer especially in high risk pregnancies.

## 1. Introduction

Labour induction in unfavourable cervix is tedious and prolonged resulting in high incidence of failed induction and hence operative deliveries. Therefore, prostaglandin E2 (PGE2, dinoprostone gel) and PGE1 (misoprostol) are commonly used for success of labour induction and to reduce rate of caesarean section [1]. Prostaglandins are quite effective for cervical ripening [2] but have a high incidence of hyperstimulation and tachysystole which may compromise the fetus [3, 4]. An ideal cervical ripening agent should ripen cervix without stimulating uterine activity.

Nitric oxide (NO) donors such as isosorbide mononitrate (IMN) and glyceryl trinitrate (GTN) effectively induce cervical ripening without causing uterine contractions by rearranging cervical collagen and ground substance which softens the cervix [5–8].

The efficacy and safety of NO donors have been established in various studies [9–12] but there has been no Indian study to compare IMN with PGE2 gel for preinduction cervical ripening in term high risk pregnancies. With this background in mind, the present study was planned to compare the efficacy and safety of IMN with PGE2 gel for cervical ripening in term pregnancy in Indian population.

## 2. Methods

A prospective, randomized study was conducted from May 9, 2011, to April 8, 2012, at Safdarjung Hospital, New Delhi, India. The protocol was approved by the ethical committee of Safdarjung Hospital, Delhi, India.

Assuming that the percentages of women with a Bishop score less than 6 after 24 hours of the initiation of the IMN and PGE2 treatment would be 40% in the IMN group and 13% in the PGE2 group [13], it was determined that a sample size of 200 women, 100 in each group, would have 80% power to detect a 27% difference between the groups at *α* = 0.05.

Study population comprised pregnant women >37 weeks hospitalised for induction of labour from outpatient department (O.P.D.) for either maternal or fetal indication. Low risk patients included postdated pregnancies (>42 weeks). High risk group included patients with hypertension, intrauterine growth restriction, cholestasis, or diabetes. All the participants had a singleton pregnancy with unfavourable cervix (Bishop score < 6), absence of uterine contractions, and intact membranes.

Exclusion criteria were fetal malpresentation, previous uterine incision, pregnancy with antepartum hemorrhage, severe anemia, heart disease or any contraindication to receive IMN or prostaglandins such as known allergy to drugs, bronchial asthma, hypotension, and palpitations.

Informed consent was obtained from all the participants. Simple randomization method was used. Patients were allocated to two groups by computer generated random numbers. The participants were enrolled by the first author and assigned to IMN and PGE2 group in accordance with the list of codes by the second author. This was a single blind trial as the participants did not know whether they were assigned to IMN or PGE2 group.

Baseline Bishop score of all the subjects was recorded. The participants were administered either 40 mg tablet of IMN (Monotrate; Sun pharmaceutical Industries, Mumbai, India) in posterior fornix which was repeated only once after 12 hours or three doses of 0.5 mg PGE2 gel (Cerviprime; Astra Zeneca Pharma India, Bangalore, India) intracervically given at 6 hours' interval.

The interval between 2 doses of vaginally administered IMN and its dosage was based on pharmacokinetics and serum profile data that revealed higher serum levels of vaginally administered 40 mg IMN tablet (337 *μ*g/L) as compared to 144 *μ*g/L with a 20 mg tablet. Cervical ripening was not improved by vaginal dose higher than 40 mg. The half-life is approximately 5 hours, the volume of distribution is 0.62 litre/kg, and systemic clearance is 115 mL/minute [14].

PGE2 is available as a 3 g endocervical gel containing 0.5 mg dinoprostone and can be repeated in 6 hours, not to exceed 1.5 mg in 24 hours. Half-life is 2.5 to 5 minutes. Onset of action is rapid and peaks in 30 to 45 minutes. *T*_max⁡_ is 0.5 to 0.75 hours. *C*_max⁡_ is approximately 484 pg/mL [15].

24 hours after first dose of IMN or PGE2, Bishop score was recorded and amniotomy done, if possible, irrespective of Bishop score. Labour was augmented by low dose oxytocin as per ACOG guidelines. Labour was immediately augmented by oxytocin if membranes ruptured spontaneously after IMN/PGE2 administration and further doses of drugs were withheld. Maternal and fetal condition and progress of labour were plotted on partogram.

Subjects who failed to achieve active phase of labour despite oxytocin stimulation for 6 hours were labelled as failed induction. Active labour was defined as at least 3 regular uterine contractions in 10 minutes, each lasting for at least 40 seconds with cervical dilatation of 3 cm or more.

The primary outcome variables were Bishop score at baseline and 24 hours after the first dose, initiation of treatment to vaginal delivery interval, and onset of active labour to vaginal delivery interval. Secondary outcome variables were subsequent need for oxytocin, operative delivery rates, and maternal side effects, that is, headache, hypotension, and complications such as hyperstimulation, tachysystole, and postpartum haemorrhage, and foetal outcome variables included abnormal foetal heart pattern, Apgar score at 1 and 5 minutes, and neonatal intensive care unit (NICU) admissions for Apgar ≤ 3 at 5 minutes of birth.

Statistical analysis was done using the following tests for comparison between IMN and PGE2 groups. Unpaired *t*-test was applied for age, gestational age, baseline Bishop score, Bishop score 24 hours after first dose, change in Bishop score, initiation of treatment to delivery interval, and labour delivery interval to see the difference between the two groups. Chi-square test has been applied for educational status, socioeconomic status, oxytocin requirement, caesarean section rate, headache, palpitation, tachysystole, hyperstimulation, postpartum haemorrhage, meconium stained liquor, nonreassuring foetal heart rate, Apgar score ≤ 3, and NICU admissions to see the association between two groups IMN and PGE2.

## 3. Results

**Figure 1:**
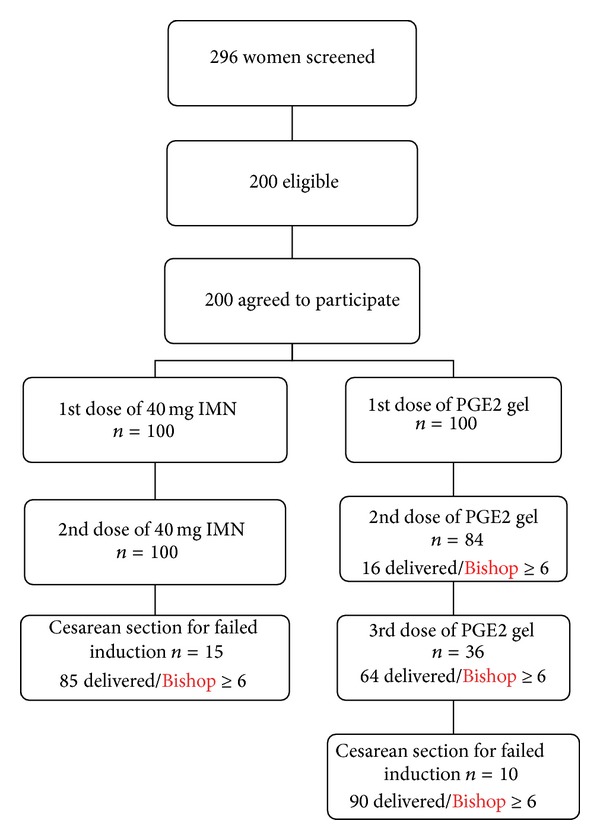
Flowchart of study participants.

A total of 200 women were recruited by computer generated random numbers, 100 to each IMN and PGE2 group (Figure 1), and simple randomization method was used. IMN group constituted 79% of women in low risk and 21% in high risk group. PGE2 group comprised 76% of patients in low risk and 24% in high risk group. Patients in low risk and high risk group were comparable in IMN and PGE2 groups. The maternal characteristics were similar in between the 2 groups (Tables 1 and 2). Most of the women were educated to more than middle school and belonged to lower middle class.

The mean Bishop score 24 hours after 1st dose of ripening agent was significantly higher in PGE2 group (*P* = 0.006). The mean change in modified Bishop score at 24 hours was 3.20 ± 1.61 for IMN and 3.87 ± 1.46 for PGE2 (*P* = 0.002; 95% CI, −1.09 to −0.24) (Table 3). The percentages of women with Bishop score < 6 at 24 hours of initiation of treatment were 35% in the IMN group and 12% in the PGE2 group (*P* < 0.001). Amniotomy was possible in all the patients. Oxytocin was required for 91% of participants in IMN group and 76% of participants in PGE2 group (*P* = 0.004). The mean time from treatment initiation to vaginal delivery was significantly shorter in PGE2 group (13.37 ± 10.67 hours) than in IMN group (30.78 ± 17.29 hours) with *P* value < 0.001 (95% CI, 12.98 to 20.99). Also, time from onset of labour to delivery interval was significantly shorter in PGE2 group (*P* < 0.001; 95% CI, 1.47 to 4.15) (Table 3).

Tachysystole was seen in a statistically significant number of patients in PGE2 group (*P* < 0.001) and 3 patients of PGE2 group had hyperstimulation whereas none of the participants in the IMN group had hyperstimulation or tachysystole (Table 4). There was no significant difference in the incidence of PPH in between the 2 groups.

A statistically significant number of patients had nonreassuring foetal heart rate pattern in the PGE2 group compared to none in the IMN group (*P* = 0.001). Nonreassuring foetal heart rate (FHR) was found in 37.5% of women in high risk group compared to 2.6% in low risk group. Also, significant number of babies had Apgar ≤ 3 at 5 minutes (*P* = 0.04) and required NICU admission in PGE2 group. Apgar ≤ 3 at 5 minutes was seen only in high risk group (Table 5).

Caesarean delivery rate was more in PGE2 group compared to IMN group (27% versus 17%) but the difference was not statistically significant (*P* = 0.22) (Table 4). After initial treatment with IMN or PGE2 followed by artificial rupture of membranes and oxytocin, 15% of participants in IMN group and 10% in PGE2 group had caesarean section for failed induction. The most common indication for caesarean section in IMN group was for failed induction (88.2%) and in PGE2 group was for fetal distress. In PGE2 group, 40.7% of caesarean section were for fetal distress compared to none in the IMN group (*P* < 0.001).

The main side effects experienced in the IMN group were headache and palpitations; however, headache was not severe and none of the patients avoided the second dose of IMN. There were no changes in basic vital signs that required treatment in either group.

## 4. Discussion

IMN and PGE2 both are effective at cervical ripening but significant improvement in mean Bishop score and less number of women with Bishop score < 6 after 24 hours of initiation of treatment, higher change in Bishop score, less oxytocin requirement, and shorter initiation of treatment to delivery interval and labour delivery interval in PGE2 group support that PGE2 is more effective than IMN.

Chanrachakul et al. [10, 11] in 2 small trials compared IMN with prostaglandin E1 (PGE1) and nitric oxide (NO) donor glyceryl trinitrate (GTN) with PGE2 gel. Both trials demonstrated that 24 hours after initiation of treatment, increase in the median Bishop score was higher in prostaglandins group compared to NO donor. Osman et al. [13] also reported significantly higher change in modified Bishop score in PGE2 group (*P* < 0.0001) and percentages of women with Bishop score < 6 requiring additional ripening agent 24 hours after initiation of treatment was found to be higher in the IMN group compared to PGE2 (40% versus 13%).

The oxytocin requirement was significantly less in PGE2 group (*P* = 0.004) compared to IMN group. Similarly, Chanrachakul et al. [10, 11] reported less oxytocin requirement in prostaglandins group compared to NO donors (75% in GTN versus 43% in PGE2 group, 92% in IMN versus 11% in PGE1 group).

In the present study, time from treatment initiation to delivery and labour delivery interval was significantly shorter in PGE2 group compared to IMN group (*P* < 0.001). Osman et al. [13] also found time from initiation of treatment to delivery interval significantly shorter in PGE2 group, 26.9 hours versus 39.7 hours in IMN group.

Tachysystole was seen in a significant number of patients in PGE2 group in the present study. There were 3 cases of hyperstimulation in PGE2 group and no case of hyperstimulation or tachysystole in IMN group. Chanrachakul et al. [10, 11] also found NO donors GTN and IMN to be less associated with uterine tachysystole compared to prostaglandins (0% in GTN versus 9% in PGE2 group, 0% in IMN versus 19% in PGE1 group). Hyperstimulation was found to be more associated with PGE1 group (0% in IMN versus 15% in PGE1 group). Sharma et al. [12] also reported hyperstimulation and tachysystole only in PGE1 (9% and 4.3%) and PGE2 groups (4.7% and 16.2%). Headache was reported in 48% of GTN subjects. In the present study, incidence of headache and palpitations was higher in IMN group compared to PGE2 group. Similar findings have also been reported in other previous studies [13, 16–18]. Headache and palpitations were more frequent with GTN and IMN group.

Statistically significant higher number of patients in the present study had nonreassuring foetal heart rate pattern in PGE2 group compared to none in IMN group. Similarly, Osman et al. [13] reported that 7% of patients had abnormal foetal heart rate patterns in PGE2 group compared to none in the IMN group.

The caesarean section rate was found to be higher in the PGE2 group than IMN (27% versus 17%) in the present study. Chanrachakul et al. [10, 11] in 2000 reported higher caesarean section rate in PGE2 group compared to IMN and GTN group (60% versus 40%).

To conclude, PGE2 is more effective than IMN for cervical ripening prior to induction of labour. However, it is associated with higher incidence of hyperstimulation, tachysystole, and nonreassuring foetal heart rate. There is only one study [13] in which IMN has been compared with PGE2 for cervical ripening at term pregnancy but they also have not categorised the results in high and low risk patients. In our study, we have compared the foetal outcome in low risk and high risk patients. Nonreassuring foetal heart rate in PGE2 group is higher in high risk group compared to low risk women. Apgar ≤ 3 at 5 minutes was seen only in high risk group in PGE2 group. Therefore, it may not be the ideal agent for cervical ripening especially in women with high risk pregnancy. IMN can be used safely in such cases.

## Figures and Tables

**Table 1:** Maternal characteristics.

Variable	IMN *N* = 100	PGE2 *N* = 100	*P* value
Age (years)^a^	23.40 ± 2.391	23.57 ± 3.655	0.69
Gestational age (weeks)^a^	42.08 ± 1.066	41.99 ± 1.218	0.57
Parity			
Nulliparous^b^	54%	51%	0.671
Multiparous^b^	46%	49%	0.671
Baseline Bishop score^a^ (pretreatment)	2.21 ± 1.241	2.20 ± 0.876	0.94

^a^Values are given as mean ± standard deviation.

^b^Values are given as percentage.

**Table 2:** Indications for induction.

Indication	IMN *N* = 100	PGE2 *N* = 100
Low risk		
Postdated	79%	76%
High risk		
Hypertension	15%	18%
Intrauterine growth restriction	3%	2%
Cholestasis	3%	4%

**Table 3:** Comparison of labour and delivery characteristics.

Variable	IMN *N* = 100	PGE2 *N* = 100	*t* value	*P* value	95% confidence interval	
					Lower	Upper
Bishop score at 24 hrs^a^ (posttreatment)	5.41 ± 1.85	6.07 ± 1.52	−2.75	0.006	−1.13	−0.18
Change in Bishop score^a^	3.20 ± 1.61	3.87 ± 1.46	−3.07	0.002	−1.09	−0.24
Labour delivery interval (hours)^a^	7.34 ± 5.51	4.53 ± 3.97	4.13	<0.001	1.47	4.15
Initiation of treatment-delivery interval (hours)^a^	30.78 ± 17.29	13.37 ± 10.67	8.36	<0.001	12.98	20.99

^a^Values are given as mean ± standard deviation.

**Table 4:** Maternal and fetal outcome.

Variables	IMN *N* = 100	PGE2 *N* = 100	Chi-square value	*P* value
Headache	46%	0%		
Not requiring medication	40%	0%	50.0	<0.001
Requiring medication	6%	0%	6.18	0.013
Palpitation	12%	0%	12.76	<0.001
Tachysystole	0%	15%	16.21	<0.001
Hyperstimulation	0%	3%	3.05	0.08
Caesarean section	17%	27%	2.95	0.22
Postpartum hemorrhage	2%	3%	0.20	0.65
Nonreassuring FHR	0%	11%	11.64	0.001
Apgar ≤ 3 at 5 min (NICU admission)	0%	4%	4.08	0.04

**Table 5:** Fetal outcome.

Risk groups	IMN *N* = 100	PGE2 *N* = 100
Low risk		
Nonreassuring FHR	0%	2/76 (2.6%)
Apgar ≤ 3 at 5 min	0%	0%
High risk		
Nonreassuring FHR	0%	9/24 (37.5%)
Apgar ≤ 3 at 5 min	0%	4/24 (16.7%)
